# Exosomal MicroRNAs Contribute to Cognitive Impairment in Hypertensive Patients by Decreasing Frontal Cerebrovascular Reactivity

**DOI:** 10.3389/fnins.2021.614220

**Published:** 2021-03-01

**Authors:** Junyi Ma, Xiang Cao, Fangyu Chen, Qing Ye, Ruomeng Qin, Yue Cheng, Xiaolei Zhu, Yun Xu

**Affiliations:** ^1^The State Key Laboratory of Pharmaceutical Biotechnology, Department of Neurology, Medical School, Drum Tower Hospital, Institute of Brain Science, Nanjing University, Nanjing, China; ^2^Jiangsu Key Laboratory for Molecular Medicine, Medical School of Nanjing University, Nanjing, China; ^3^Jiangsu Province Stroke Center for Diagnosis and Therapy, Nanjing, China; ^4^Nanjing Neurology Clinic Medical Center, Nanjing, China

**Keywords:** hypertension, exosomal microRNA, cerebrovascular reactivity, mediation, cognitive impairment

## Abstract

Mechanisms underlying cognitive impairment (CI) in hypertensive patients remain relatively unclear. The present study aimed to explore the relationship among serum exosomal microRNAs (miRNAs), cerebrovascular reactivity (CVR), and cognitive function in hypertensive patients. Seventy-three hypertensive patients with CI (HT-CI), 67 hypertensive patients with normal cognition (HT-NC), and 37 healthy controls underwent identification of exosomal miRNA, multimodal magnetic resonance imaging (MRI) scans, and neuropsychological tests. CVR mapping was investigated based on resting-state functional MRI data. Compared with healthy subjects and HT-NC subjects, HT-CI subjects displayed decreased serum exosomal miRNA-330-3p. The group difference of CVR was mainly found in the left frontal lobe and demonstrated that HT-CI group had a lower CVR than both HT-NC group and control group. Furthermore, both the CVR in the left medial superior frontal gyrus and the miRNA-330-3p level were significantly correlated with executive function (*r* = −0.275, *P* = 0.021, and *r* = −0.246, *P* = 0.04, respectively) in HT-CI subjects, and the CVR was significantly correlated with the miRNA-330-3p level (*r* = 0.246, *P* = 0.040). Notably, path analysis showed that the CVR mediated the association between miRNA-330-3p and executive function. In conclusion, decreased miRNA-330-3p might contribute to CI in hypertensive patients by decreasing frontal CVR and could be a biomarker of early diagnosis.

## Introduction

As a worldwide public health problem, hypertension is detected in 31.1% of adults worldwide (Mills et al., 2016). It has been reported that hypertension is one of the major risk factors for dementia. There is a strong correlation between hypertension and the progression of dementia (Li et al., 2016). High blood pressure has been correlated with multiple cognitive dysfunctions such as executive dysfunction, reduced mental processing speed, as well as memory deficit (Gąsecki et al., 2013). A 40-year follow-up study of a population-based cohort investigated the association of vascular risk factors with all types of dementia and found that high systolic blood pressure was consistently associated with all types of dementia (Ronnemaa et al., 2011). In a 17-year observational study of 668 non-demented elderly Japanese, subjects with hypertension had at least three-fold greater risk of vascular dementia compared to subjects with normal blood pressure (Ninomiya et al., 2011).

Hypertension is thought to affect the structure and function of cerebral blood vessels. There are multiple adaptive changes in the extravascular environment, local signaling molecules, or hemodynamic demands in the healthy arteries to keep blood pressure homeostasis, but these adaptive changes do not return to baseline and induce pathological vascular alterations in disease states. Multiple cellular components such as vascular smooth muscle cells (VSMCs) in the vessel wall of both large and small arteries, endothelial cells, and elastin and collagen content were changed in hypertension (Wong et al., 2010; Savoia et al., 2011; Wagenseil and Mecham, 2012; Duca et al., 2016). With the development of chronic hypertension, the structure of arterial wall has remodeled, such as thickening, stiffening, and narrowing, which are reactions to the stress of arterial walls (Wilstein et al., 2018). However, how these hypertension-related cerebrovascular alterations result in cognitive impairment (CI) remains relatively unknown. Cerebrovascular reactivity (CVR) is a vasodilatory index that is related to the important function of dilation and constriction of cerebral vessels in response to changes of resource requirement in vessel system. Compared with other cerebral vascular indexes such as cerebral blood flow (CBF), CVR is regarded as a more specific vascular signal (Liu et al., 2017). Two major techniques in testing cerebrovascular reactivity are functional magnetic resonance imaging (fMRI) and transcranial Doppler sonography (Grant et al., 2015). fMRI represents a unique and noninvasive technique in measuring cerebral vascular state. Blood oxygen level-dependent (BOLD) signal, detected with fMRI technique, is equipped with the advantage of high signal-to-noise ratio, which is capable of measuring CVR (Kannurpatti et al., 2014). Previous studies have suggested that BOLD-based fMRI signal is basically adjusted by physiology of vessels (Kannurpatti et al., 2010; Liu et al., 2013). Reduced CVR has been observed in specific regions of the brain in hypertensive patients. However, the molecular mechanism underlying the altered CVR and the relationship between CVR and cognitive function in hypertensive patients are relatively unclear.

Exosomes are small extracellular membrane vesicles with a diameter of 30–150 nm. They are secreted by all living cells and contain microRNAs (miRNAs), DNA, lipid, protein, and mRNAs (Chopp and Zhang, 2015; Mathieu et al., 2019). Exosomes exist in different types of body fluid, including saliva, blood, and urine, and serve as important carriers that transfer both proteomic and genomic materials between cells. As a kind of endogenous and small non-coding RNAs, miRNAs play a critical role in restraining target RNAs’ translation or facilitating decomposition of target RNAs (Bartel, 2004). MiRNAs are more stable in serum exosomes compared with those free in serum due to the features of exosomes to protect the structure and biological activity of miRNA from RNase digestion. Therefore, specific serum exosomal miRNAs are regarded as novel regulators of neural behavior not only in protein translation but also in inflammatory processing (Wood et al., 2011).

Plentiful studies have shown that miRNA levels related to neurological insult are significantly changed in essential hypertension and secondary hypertension (Shi et al., 2015). MiRNAs are involved in the pathogenesis of hypertension through a variety of mechanisms, including the regulations of endothelial function, nitric oxide-dependent vasodilatation, and sympathetic activity (Klimczak et al., 2017). Specifically, altered circulating miRNAs including miRNA-1, 21, 133, 145, 505, and 510 have been strongly associated with the pathogenesis and progression of essential hypertension (Kontaraki et al., 2014; Yang et al., 2014; Krishnan et al., 2017). MiRNAs regulate a series of vascular processes, such as angiogenesis, apoptosis, proliferation, and migration by targeting specific mRNA (Ballantyne et al., 2016), and most of the processes are related to endothelial cell function. Moreover, endothelial dysfunction is considered as an important contributor to the imbalance between vasoconstrictors and vasodilators in the pathogenesis of hypertension (Widlansky et al., 2003; Nemecz et al., 2016; Zhang et al., 2018). Since CVR serves as a specific measurement for the dilation and constriction of cerebral vessels, abnormal miRNA might lead to altered CVR in hypertensive patients.

The present study recruited hypertensive patients with cognitive impairment (CI), hypertensive patients with normal cognition, and healthy subjects. All subjects underwent identification of exosomal miRNA, multimodal MRI scans, and neuropsychological tests. We explored the patterns of exosomal miRNA, CVR maps, and cognitive function in hypertensive patients and the relationship among them. We also explored whether there were mediating effects of CVR on the relationship between miRNA and CI in hypertensive patients with CI. We hypothesized that CI of hypertensive patients would be related to abnormal exosomal miRNA and altered CVR patterns, and CVR might mediate the link between exosomal miRNA and CI.

## Materials and Methods

### Participants

Individuals were recruited from the outpatients and inpatients of the Department of Neurology of Nanjing Drum Tower Hospital between January 2017 and June 2019. The present study was approved by the Nanjing Drum Tower Hospital Research Ethics Committee. All participants provided written informed consent ahead of participation. Inclusion criteria for the hypertension group were as follows: (1) ≥50 years of age and (2) classified as hypertension by experienced physician based on standard guidelines (systolic blood pressure (SBP) >140 mmHg or/and diastolic blood pressure (DBP) >90 mmHg) (Mancia et al., 2013), with/without CI. The control group was selected from participants with normal blood pressure and cognition. The CI of participants was evaluated based on the standard of our previous study, and the cut-off was based on the Mini Mental State Examination (MMSE)/Montreal Cognitive Assessment (MoCA) scores and educational experience of the subjects (Gu et al., 2019). The participants with scores less than the cut-off (for MMSE score, the cut-off was ≤26 for >6 educational years, ≤22 for 1–6 educational years, and ≤19 for 0 educational years, and for MoCA score, the cut-off was ≤25 for >12 educational years, ≤24 for 7–12 educational years, ≤19 for 1–6 educational years, and ≤13 for 0 educational years) were defined as cognitive impairment (Table 1). Exclusion criteria were as follows: (1) severe chronic diseases (e.g., cardiac insufficiency, renal insufficiency, cancer, severe anemia, shock, systemic lupus erythematosus, or thyroid dysfunction); (2) anxiety, depression, or other mental diseases; (3) contraindications of MRI scanning or obvious motion artifacts on any image series; (4) a history of Alzheimer’s disease, Parkinson’s disease, multiple sclerosis, neuromyelitis optica, or epilepsy; (5) severe carotid artery or vertebral stenosis; and (6) severe impaired vision or audition.

**TABLE 1:** The evaluation standard of cognitive impairment.

	MMSE			MoCA			
Education (years)	0	1–6	>6	0	1–6	7–12	>12
Threshold value of cognitive impairment (score)	≤19	≤22	≤26	≤13	≤19	≤24	≤25

*The subjects with scores equal to or lower than the threshold in MMSE or MoCA tests in different educational experience layers were defined as cognitive impairment. MMSE, Mini-Mental State Examination; MoCA, Montreal Cognitive Assessment.*

A total of 177 subjects were recruited, namely, 73 hypertensive patients with CI (HT-CI), 67 hypertensive patients with normal cognition (HT-NC), and 37 healthy controls. All subjects underwent MRI examination, serum collection, neuropsychological tests, and B-mode ultrasonography of the carotid and vertebral arteries or coronal computed tomography angiography (CTA).

### Neuropsychological Examination

Neuropsychological tests were performed by a professional neuropsychologist on the day of MRI scanning. Six domains of cognitive function were evaluated, including global cognition, processing speed, memory, executive function, visuospatial function, and language. All the raw scores were transformed into *Z* scores according to means and standard deviation (SD) of the row scores: ($Z=\frac{X-\bar{X}}{\text{SD}}$), and $\bar{X}$ indicates mean of raw score. MMSE and MoCA were used to evaluate general cognition. The mean of *Z* scores of Visual Reproduction-delayed recall test (VR-DR) and the Auditory Verbal Learning Test-long time delayed recall test (AVLT-DR) were used to assess memory. Processing speed was examined via the average *Z* scores of Trail Making Test A (TMT-A) and Stroop Color and Word Test (SCWT) A. Executive function was evaluated from the mean *Z* scores from Category Verbal Fluency (CVF), Trail Making Test A and B, and the Stroop-C test. Language was assessed using the average *Z* scores from Boston Naming Test (BNT) and the Category Verbal Fluency. In addition, visuospatial ability was evaluated using the average *Z* scores of Visual Reproduction (VR)-copy and the clock-drawing test (CDT).

### Serum Collection

Venous blood samples were collected in vacutainer tubes in the morning and then were centrifuged at 3,000 rpm for 10 min. The serum was transferred to a fresh Eppendorf tube and centrifuged at 10,000 rpm for another 10 min to eliminate cellular debris and red blood cells.

### Isolation and Characterization of Exosomes

To separate exosomes from serum, ExoQuick serum prep and exosome precipitation kit (System Biosciences Inc., Mountain View, CA, United States) was used according to the manufacturer’s protocol. Briefly, a one-fourth volume of exosome precipitation solution was added to the serum and mixed gently by inverting the tube and then the sample was refrigerated for 30 min at 4°C. After centrifuging (1,500 *g*) for 30 min, the supernatant was removed carefully and the pellets were resuspended in sterile PBS. Exosomes were visualized by transmission electron microscopy (TEM) and detected on a nanoparticle-tracking analyzer (NTA; Malvern Panalytical, Malvern, United Kingdom) as described previously (Ji et al., 2016).

### Western Blotting Analysis

Exosomes were lysed with lysis buffer (Thermo Fisher Scientific, Rockford, IL, United States) for 30 min at 4°C. The concentration was measured via BCA methods after centrifuging at 12,500 rpm for 30 min. Equal quantities of proteins were separated with 10% SDS-PAGE and then transferred onto PVDF membranes (Millipore, Billerica, MA, United States). After blocking with 5% slim milk for 1 h, the membranes were incubated with primary antibodies against Alix, CD54, Flotillin-1, CD9, and CD63, which are specific proteins of exosomes (Cell Signaling Biotechnology, Hertfordshire, England). The proteins were scanned with a Gel-Pro system (Tanon Technologies, Shanghai, China).

### RNA Extraction and Quantitative Reverse Transcription Polymerase Chain Reaction

An exosome RNA Purification Kit (System Biosciences Inc) was used to extract total RNA from serum exosomes following the manufacturer’s conditions. RNA was quantified using an Agilent Bioanalyzer 2100 (Santa Clara, CA, United States). Then, the isolated RNA was reverse transcribed using a miRNA Reverse Transcription Kit (Thermo Fisher Scientific, Rockford, IL, United States) according to the following protocols: 16°C for 30 min, 42°C for 30 min, and 85°C for 5 min. Quantitative reverse transcription polymerase chain reaction (qRT-PCR) was carried out on a StepOne Plus Real-Time PCR System (Applied Biosystems, Foster City, United States) with TaqMan Universal Master Mix (Thermo Fisher Scientific). The expression levels of selected miRNAs detected by qRT-PCR were normalized to spiked-in *Caenorhabditis elegans* cel-miR-39 (RiboBio, Guangzhou, China) and the data were analyzed using the 2^–Δ*Ct*^ (ΔCt = CtmiRNA − CtmiR39) method.

### Exosomal miRNA Profile

MiRNA deep sequencing was performed from the serum of the most typical subjects, including six HT-NC patients, six HT-CI patients, and three control individuals in Nanjing Geneseeq Inc., Nanjing, China. Briefly, serum exosomal total RNA quality was assessed using Agilent Bioanalyzer 2100. Then, the sequencing libraries were generated using NEBNext Multiplex Small RNA Library Prep Set (New England Biolabs, United States) following manufacturer’s recommendations, and index codes were added to attribute sequences to each sample. Libraries were pooled and sequenced on an Illumina X-ten PE150 platform. A minimum of 300 M reads was generated per sample.

### MRI Acquisition

All experiments were performed on a Philips Ingenia 3.0T scanner (Philips, Eindhoven, Netherlands), equipped with a 32-channel head coil. All the images were collected through a same MRI machine to keep homogeneity of images. Head motion was minimized using foam padding, and scanner noise were reduced using a pair of earplugs. Resting-state functional imaging was obtained using a gradient-echo echo-planar imaging (GRE-EPI) sequence, and the imaging parameters were as follows: repetition time (TR) = 2000 ms, flip angle (FA) = 90°, echo time (TE) = 30 ms, matrix = 64 × 64, thickness = 4.0 mm, voxel size = 3 × 3 × 3 mm, field of view = 192 × 192 mm, number of slices = 35, and gap = 0 mm. The scan process totally continued for 8 min and 7 s, and subjects received an instruction to keep still during the whole procedure. The three-dimensional T1-weighted sagittal imaging were acquired by turbo fast echo sequence: TR = 9.8 ms, FA = 8°, TE = 4.6 ms, matrix = 256 × 256, thickness = 1.0 mm, voxel size = 1 × 1 × 1 mm, field of view = 256 × 256 mm, number of slices = 192, and gap = 0 mm. The three-dimensional FLAIR sagittal imaging was acquired by the following sequence: TR = 4,500 ms, FA = 90°, TE = 344 ms, matrix = 272 × 272, thickness = 1.0 mm, number of slices = 200, and gap = 0 mm.

### MRI Processing

Data were analyzed using the toolbox for Statistical Parametric Mapping (SPM) and Data Processing and Analysis for Brain Image (DPABI 4.0)^1^ software, which are performed in MATLAB (MathWorks, Natick, MA, United States). Preprocessing of the BOLD image series was performed including transformation from DICOM to NIFIT format, head motion correction, smoothing with a Gaussian filter of an 8-mm full-width half-maximum (FWHM), and linear detrending. The end-tidal CO_2_ (EtCO_2_) time course was shifted using a step-wise searching procedure mentioned previously to figure up the time for the blood travel from the lung to the brain. Afterward, the shifted EtCO_2_ time course was in sync with BOLD imaging and was used for the following analysis. Previous study has shown that the correlation between EtCO_2_ and BOLD signal time courses was highest when the time course was at the 0.02–0.04 Hz frequency range (Liu et al., 2017); thus, the BOLD signal was filtered at the frequency range of 0.02–0.04 Hz. The average whole-brain BOLD time course was obtained using the whole-brain mask and was used as a reference signal. Then, the CVR index was calculated by employing a general linear model with an independent variable of the reference BOLD signal and dependent variable of the voxel’s signal time course and was normalized to the reference region values, yielding a relative CVR map. Relative CVR map was obtained in individual space and then normalized to Montreal Neurological Institute (MNI) standard space with the resample size set at 3 mm × 3 mm × 3 mm. Voxel-wise group comparison and ROI relative CVR value extraction based on the group comparison were performed on DPABI. Automated white matter hyperintensity (WMH) was performed using a brain quantification tool AccuBrain (Wang et al., 2019). The preprocessing steps included noise reduction, bias field correction, and intensity normalization, which was used to normalize intensity level of MRIs acquired from multiple MRI scanners. The segmentation and quantification of WMH are based on a deep learning framework on T1 and FLAIR images, which was trained on hundreds of MRI acquired from different individuals using different scanners and with highly variable degrees of hyperintensity.

### Statistical Analysis

Statistical evaluation was conducted using the software SPSS 22.0. For the first analysis, we investigated demographic data in one-way analysis of variance (ANOVA) or chi-square test. As the data of miRNA concentrations were not normally distributed, log transformation was made in miRNA data. The neuropsychological data and miRNA data were performed by covariance analysis, controlling for age, years of education, and history of lacunar stroke. The second analysis focuses on the CVR, and all the BOLD data were analyzed by DPABI 4.0. The analysis of covariance (ANCOVA) was conducted on individual normalized maps in a voxel-wise manner within a whole-brain mask to analyze the differences of CVR among the three groups, controlling for the age, years of education, and history of lacunar stroke. The threshold was determined using Gaussian random field (GRF) for multiple comparisons in the whole brain (voxel-wise *p*-value < 0.005, cluster *p*-value < 0.001, and cluster size >20 voxels). The average CVR significant regions of interest were extracted in every subgroup. Then, the mean CVR in each significant cluster was extracted in each subject. A *post hoc t*-test was performed to detect the detailed group CVR difference in each region using the SPSS 22.0 software. Finally, mediation analysis was performed on the question whether miRNA mediates the relationship between CVR and cognitive performance, which was conducted in PROCESS, controlling for age, years of education, and history of lacunar stroke. The significance was set at *p* < 0.05.

## Results

### Demographic, Neuropsychological Characteristic, and WMH Volumes

The HT-NC group, the HT-CI group, and the control group did not significantly differ in gender, the ratio of diabetes, hyperlipidemia, smoking, or drinking. However, the control group was significantly younger than the two hypertensive groups. The educational year of the HT-CI group was less than that of the HT-NC group and the control group. Moreover, the ratio of the history of lacunar stroke in the HT-CI group was higher than that in the HT-NC group and the control group (Table 2). The results of covariance analysis of cognitive performance and WMH volumes are shown in Table 2, controlling for age, years of education, and history of lacunar stroke. The HT-CI group was significantly worse than the HT-NC group and control group in all six domains of cognition. The difference of WMH volume between groups has no significance.

**TABLE 2:** Demographic, neuropsychological, and volume data.

Items	Control (*N* = 37)	HT-NC (*N* = 67)	HT-CI (*N* = 73)	*F* or χ^2^	*P*
Sex, male/female	19/18	38/29	37/36	0.568	0.753
Age, years	58.51 ± 7.194	63.85 ± 8.214^*a*^	66.03 ± 8.197^*a*^	10.853	< 0.001***
Education, years	12.95 ± 4.453	11.97 ± 3.622	11.07 ± 3.634^*ab*^	3.080	0.048*
Lacunar stroke, *n* (%)	6 (16)	13 (19)	26 (36)^*ab*^	6.935	0.031*
Diabetes, *n* (%)	5 (14)	16 (24)	16 (22)	1.627	0.443
Coronary disease, *n* (%)	1 (3)	2 (3)	6 (8)	2.533	0.282
Hyperlipidemia, *n* (%)	7 (19)	16 (24)	15 (21)	2.127	0.712
Smoking, *n* (%)	8 (22)	17 (25)	13 (18)	1.186	0.553
Alcohol, *n* (%)	10 (27)	16 (24)	12 (16)	2.005	0.367
Global cognition	0.52 ± 0.56	0.39 ± 0.55	−0.62 ± 0.87^*ab*^	39.409	< 0.001***
Processing speed	0.35 ± 0.70	0.18 ± 0.83	−0.35 ± 0.78^*ab*^	5.138	0.008**
Executive function	0.15 ± 0.59	0.13 ± 0.53	−0.20 ± 0.50^*ab*^	4.708	0.010*
Memory	0.32 ± 0.68	0.14 ± 0.63	−0.30 ± 0.96^*ab*^	3.746	0.026*
Language	0.43 ± 0.71	0.18 ± 0.74	−0.41 ± 0.82^*ab*^	13.407	< 0.001***
Visuospatial function	0.14 ± 0.64	0.26 ± 0.31	−0.32 ± 1.12^*ab*^	7.893	0.001**
WMH volume (ml)	1.49 ± 1.58	4.37 ± 6.38	5.61 ± 10.17	0.934	0.395

Data are represented as mean ± SD or *n* (%). One-way ANOVA was performed on normal distributed data. χ ^2^ analysis was performed on sex data. Covariance analysis was performed on the comparison of *Z* scores in global cognition, processing speed, executive function, memory, language, visuospatial function, and WMH volume data, controlling for age, years of education, and history of lacunar stroke. ^*a*^*P* < 0.05, compared with control group. ^*b*^*P* < 0.05, compared with HT-NC group. *P < 0.05, **P < 0.01, ***P < 0.001. HT-NC, hypertensive patients with normal cognition; HT-CI, hypertensive patients with cognitive impairment.

### Morphological and Biochemical Characterization of the Exosomes From Patients

**FIGURE 1:**
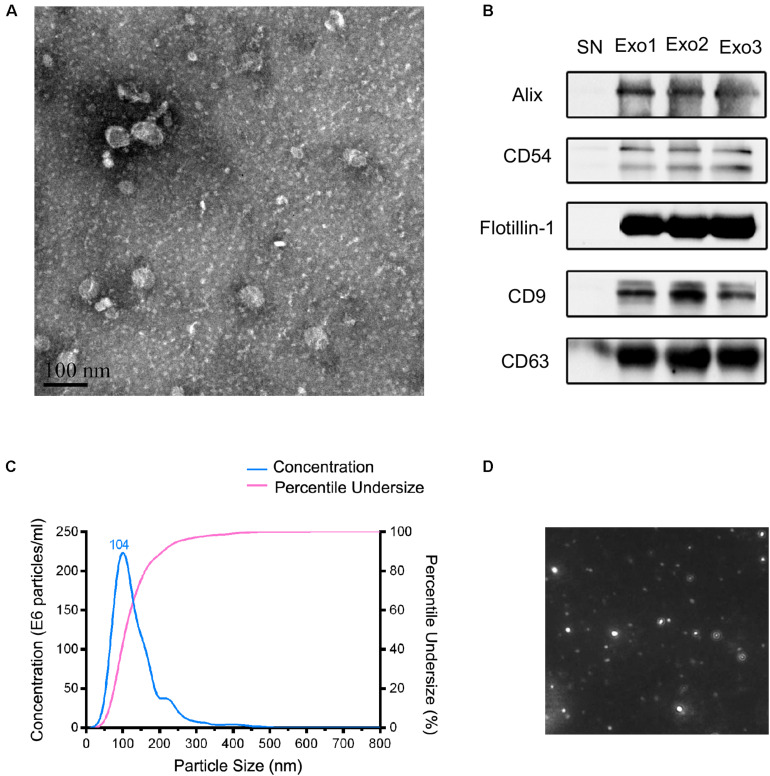
Characteristics of the obtained exosomes from human serum in this study. **(A)** The transmission electron microscopy of the exosomes from the serum. Scale bar = 100 nm. **(B)** Identification of exosomal markers with indicated antibodies by western blot. Measurement of size distribution **(C)** and morphology **(D)** of exosomes purified from serum by nanoparticle-tracking analyzer.

The isolated exosomes were characterized by TEM, nanoparticle tracking analysis, and western blotting. TEM analysis indicated that the serum micro-vesicles from patients exhibited a typical round or “cup-shape” appearance (Figure 1A). As shown in Figure 1B, the exosome markers including Alix, CD54, Flotillin-1, CD9, and CD63 were detected in the serum exosomes by western blotting. The main peak size was within the exosome particle size range (30–150 nm, Figures 1C,D). Taken together, these results confirmed that the observed exosomes showed the majority characteristics of exosomes through our isolation operation.

### Discovery of Candidate Exosomal MiRNA in the Screen Stage

**FIGURE 2:**
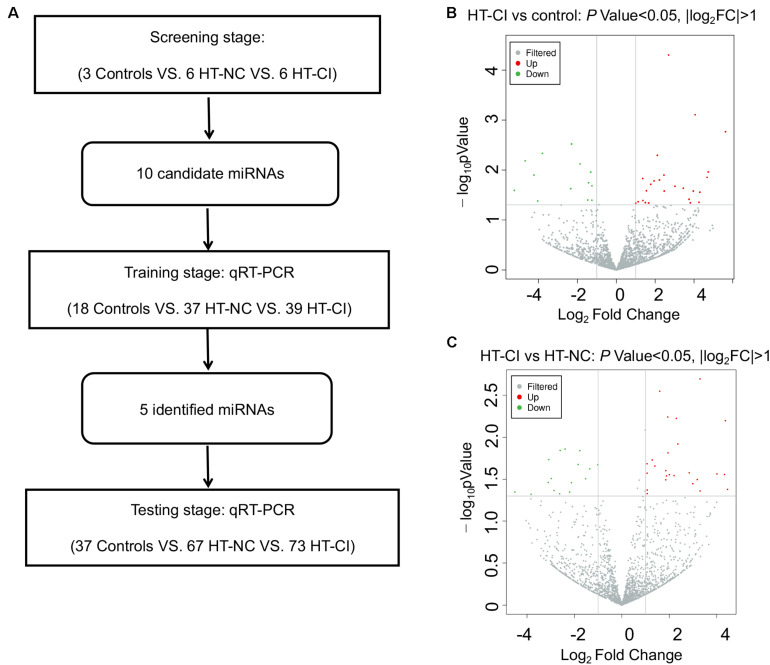
Screening on differentially expressed miRNA. **(A)** Flow chart of study design. **(B)** Volcano plot of differentially expressed miRNAs between HT-CI and control groups. **(C)** Volcano plot of differentially expressed miRNAs between HT-CI and HT-NC groups. HT-NC, hypertensive patients with normal cognition; HT-CI, hypertensive patients with cognitive impairment.

Three stages, including screening, training, and validation, were performed to define potential serum exosome miRNA biomarkers for hypertensive patients with CI (Figure 2A). Deep sequencing was used on the isolated exosomal miRNA from HT-NC and HT-CI patients and corresponding controls. The results showed that there were 2,489 common and novel miRNAs in all samples. There were a total of 25 elevated exosomal miRNAs and 14 downregulated exosomal miRNAs in the HT-CI groups as compared to controls (Figure 2B). Compared to the HT-NC group, 42 miRNAs were changed (25 miRNAs were elevated and 17 miRNAs were downregulated) (Figure 2C). Among them, nine miRNAs were elevated (miRNA-107, miRNA-191-3p, miRNA-223-3p, miRNA-330-3p, miRNA-339-3p, miRNA-432-5p, miRNA-625-3p, miRNA-671-3p, and miRNA-7641) and one miRNA was downregulated (miRNA-6852-3p) in the HT-CI group as compared to the control group and the HT-NC group.

### Validation of Candidate Exosomal MiRNA by qRT-PCR

**FIGURE 3:**
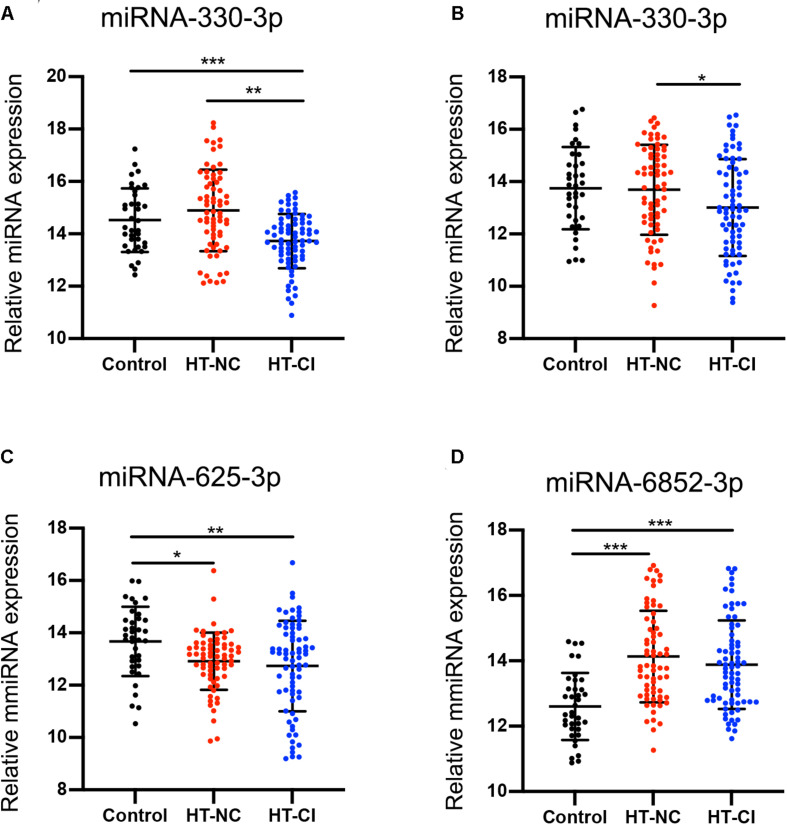
MicroRNA difference among the three groups. **(A)** Subjects in the HT-CI group demonstrated significantly lower concentration of miRNA-330-3p than the other two groups. **(B)** Subjects in the HT-CI group displayed significantly lower concentration of miRNA-432-5p than the HT-NC group. **(C)** Subjects in the HT-CI group and HT-NC group showed significantly lower miRNA-625-3p than the control group. **(D)** Subjects in the HT-CI group and HT-NC group showed significantly higher miRNA-6852-3p than the control group. Error bar indicates standard deviation. *P < 0.05, **P < 0.01, ***P < 0.001. HT-NC, hypertensive patients with normal cognition; HT-CI, hypertensive patients with cognitive impairment.

To determine the accuracy of the miRNA sequencing results, we validated the expression of exosomal miRNAs by qRT-PCR. The 10 candidate miRNAs were firstly analyzed from 37 HT-NC patients, 39 HT-CI patients, and 18 controls in the training stage. Only five miRNAs (miRNA-330-3p, miRNA-339-3p, miRNA-432-5p, miRNA-625-3p, and miRNA-6852-3p) showed consistent up- or downregulation tendency (Supplementary Figures 1A–E). In contrast, no significant differences of miRNA-107, miRNA-191-3p, miRNA-223-3p, miRNA-671-3p, and miRNA-7641 were observed among HT-NC, HT-CI, and control groups (Supplementary Figures 1F–J). The difference of miRNAs among three groups was further measured in a larger cohort of 30 HT-NC patients, 34 HT-CI patients, and 19 control serum samples using covariant analysis in validation stage. The result is shown in Figure 3, controlling for age, years of education, and history of lacunar stroke. There were four miRNAs screened out to be related to hypertension and CI, including miRNA-330-3p, miRNA-432-5p, miRNA-625-3p, and miRNA-6852-3p (Figure 3). *Post hoc* analysis showed that the HT-CI group had lower miRNA-330-3p and miRNA-625-3p than the control group and had lower miRNA-330-3p and miRNA-432-5p than the HT-NC group. Furthermore, both the HT-NC group and HT-CI group had higher miRNA-6852-3p than the control group.

### Bioinformatics Analysis of Identified Exosomal MiRNAs

**FIGURE 4:**
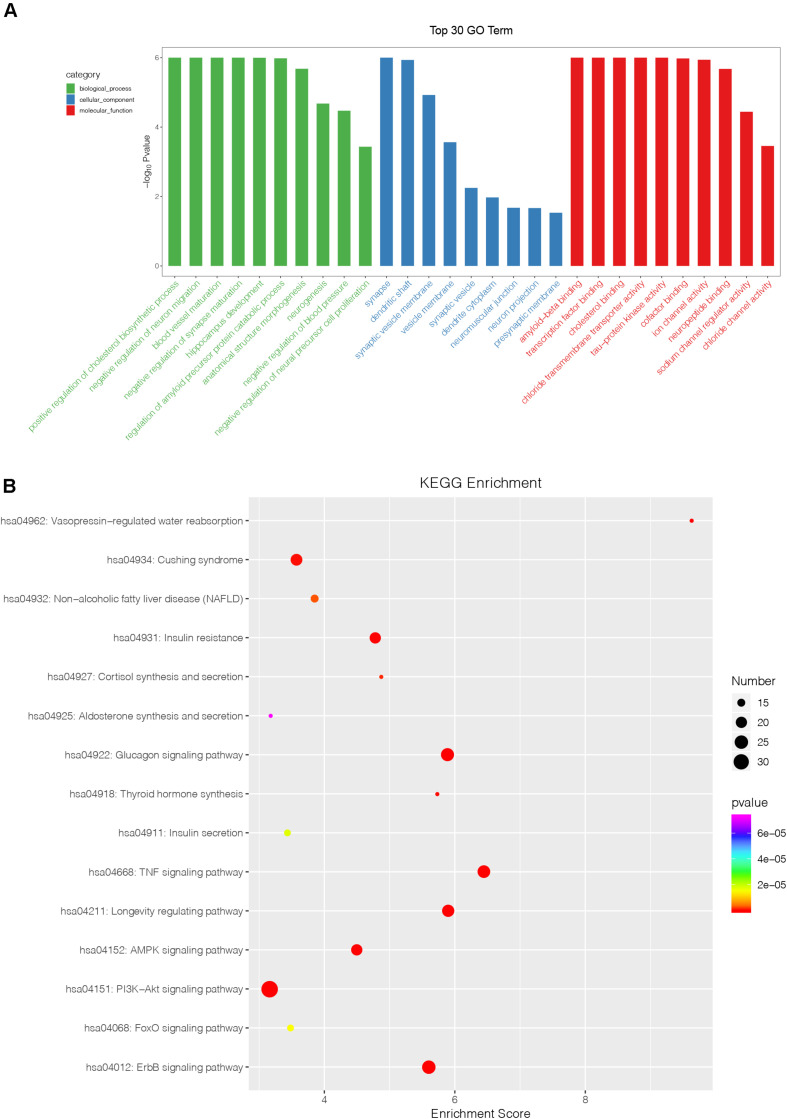
Bioinformatics analysis of identified miRNAs. **(A)** The results of Gene Ontology function analysis on differentially expressed miRNA target genes. **(B)** The entries of Kyoto Encyclopedia of Genes and Genomes pathway enrichment analysis on differentially expressed miRNA target genes.

To investigate the potential role of four identified exosomal miRNAs, we identified the target genes of the differentially expressed miRNAs with miRDB, miRanda, and TargetScan software. It was found that there were 202 target genes recruited. Then, Gene Ontology (GO) category analysis and Kyoto Encyclopedia of Genes and Genomes (KEGG) analysis were used to elucidate the biological function of these target genes. GO category analysis identified 676 biological process (BP) entries, 174 cellular component (CC) entries, and 251 molecular function (MF) entries, respectively (Figure 4A). Positive regulation of cholesterol biosynthetic process, negative regulation of synapse maturation, and blood vessel maturation are the top three enriched BP terms. The top enriched CC and MF were synapse and amyloid-beta binding, respectively. According to the KEGG pathway enrichment analysis, our identified four exosomal miRNAs were involved in several inflammation and proliferation-related pathways, such as glucagon signaling pathway, TNF signaling pathway, ErbB signaling pathway, PI3K-Akt signaling pathway, and FoxO signaling pathway (Figure 4B).

### CVR Data

**FIGURE 5:**
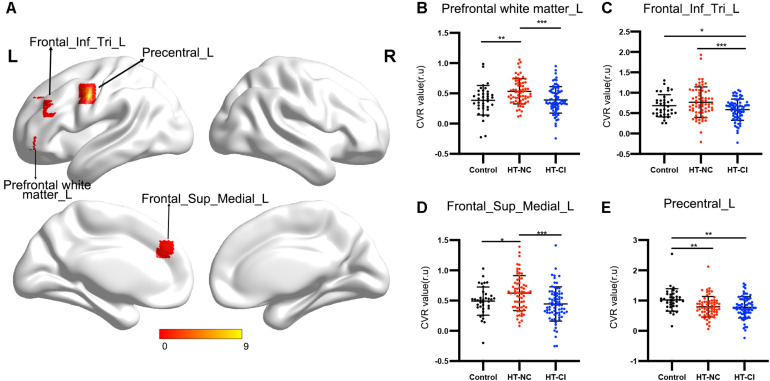
The CVR difference among the three groups. **(A)** Significant CVR regions on the brain. The four regions with significant difference were all located in the left frontal lobe. **(B)** Subjects in the HT-NC group demonstrated significantly higher CVR in the left prefrontal white matter than the other two groups. **(C)** Subjects in the HT-CI group demonstrated significantly lower CVR in the left triangular inferior frontal gyrus. **(D)** Subjects in the HT-NC group demonstrated significantly higher CVR in the left medial superior frontal gyrus than the other two groups. **(E)** Subjects in the HT-CI group and HT-NC group showed significantly lower CVR in the left precentral gyrus than the control group. The threshold was set at corrected *P*-value < 0.005, determined by Gaussian random field for multiple comparisons. Error bar indicates standard deviation. *P < 0.05, **P < 0.01, ***P < 0.001. Prefrontal white matter_L, left prefrontal white matter; Frontal_Inf_Tri_L, left triangular inferior frontal gyrus; Frontal_Sup_Medial_L, left medial superior frontal gyrus; Precentral_L, left precentral gyrus; r.u., relative unit.

The comparison of CVR variability among the three groups is shown in Figure 5. After adjustment for age, years of education, and history of lacunar stroke, significant differences of CVR were mainly found in the left prefrontal white matter, left triangular inferior frontal gyrus, left medial superior frontal gyrus, and left precentral gyrus (Table 3). *Post hoc* analysis showed the following: Firstly, significant differences of CVR were shown between HT subjects and control subjects. Compared with the control group, both the HT-NC group and the HT-CI group displayed decreased CVR in the left precentral gyrus. Secondly, the HT-CI group displayed lower CVR than the HT-NC group in the left prefrontal white matter, left triangular inferior frontal gyrus, and left medial superior frontal gyrus. Finally, compared with the control group, the HT-NC group displayed increased CVR in the left prefrontal white matter and left medial superior frontal gyrus.

**TABLE 3:** Clusters with significant difference in the cerebrovascular reactivity among groups.

	BA	Volume	Peak MNI	*F*-value
		(mm^3^)	coordinate (mm)	
			*X*, *Y*, *Z*	
Prefrontal white matter_L	11.47	117	−24, 39, 0	8.866
Frontal_Inf_Tri_L	48	21	−36, 30, 24	6.7435
Frontal_Sup_Medial_L	32	39	−15, 39, 30	7.9402
Precentral_L	6	28	−48, −6, 42	6.7351

*(X, Y, Z) coordinates of primary peak locations in the MNI space. BA, Brodmann area; MNI, Montreal Neurological Institute; Prefrontal white matter_L, left prefrontal white matter; Frontal_Inf_Tri_L, left triangle inferior frontal gyrus; Frontal_Sup_Medial_L, left medial superior frontal gyrus; Precentral_L, left precentral gyrus.*

### The Relationship Between CVR, Exosomal MiRNA, and Cognition in Hypertensive Patients With Cognitive Impairment

**FIGURE 6:**
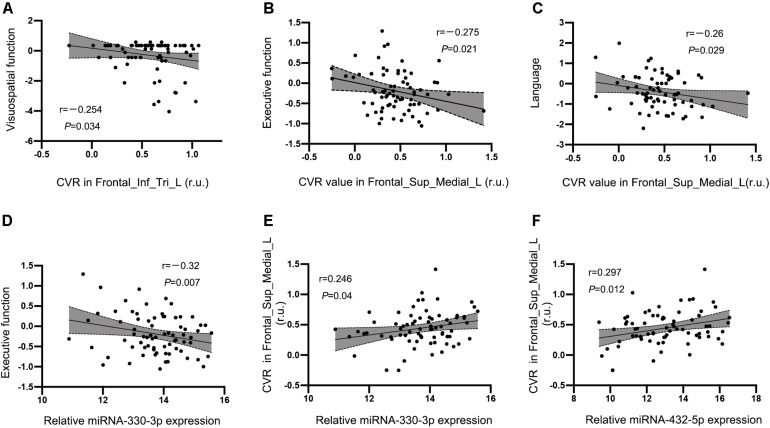
Correlations between CVR, miRNA, and cognitive performance in the HT-CI group. **(A)** The CVR value in the left triangular inferior frontal gyrus was negatively correlated with visuospatial function. **(B,C)** The CVR of the left medial superior frontal lobe had a negative correlation with executive function and language. **(D)** A significant inverse correlation between serum miRNA-330-3p and executive function was discovered in the HT-CI group. **(E,F)** The CVR of the left medial superior frontal lobe had a positive correlation with serum miRNA-330-3p and miRNA-452-5p. Frontal_Inf_Tri_L, left triangular inferior frontal gyrus; Frontal_Sup_Medial_L, left medial superior frontal gyrus; r.u., relative unit.

No significant correlation between CVR in the left prefrontal white matter or left precentral gyrus and cognition were observed in the HT-CI group. The CVR value in the left triangular inferior frontal gyrus was negatively correlated with visuospatial function (*r* = −0.254, *P* = 0.034) (Figure 6A). The correlation was also observed between CVR in the left medial superior frontal gyrus and executive function (*r* = −0.275, *P* = 0.021) (Figure 6B) or language (*r* = −0.26, *P* = 0.029) (Figure 6C), after adjustment for age, years of education, and history of lacunar stroke.

A significant correlation between serum miRNA-330-3p and executive function was found in the HT-CI group (*r* = −0.32, *P* = 0.007) (Figure 6D). There was no correlation between cognitive performance and other serum miRNA (miRNA-432-5p, miRNA-6852-3p, and miRNA-625-3p).

The CVR in the region of interest of the left medial superior frontal lobe was associated with the levels of exosomal miRNAs including miRNA-330-3p (*r* = 0.246, *P* = 0.040) (Figure 6E) and miRNA-432-5p (*r* = 0.297, *P* = 0.012) (Figure 6F) after controlling for age, years of education, and history of lacunar stroke.

### Path Analysis

**FIGURE 7:**
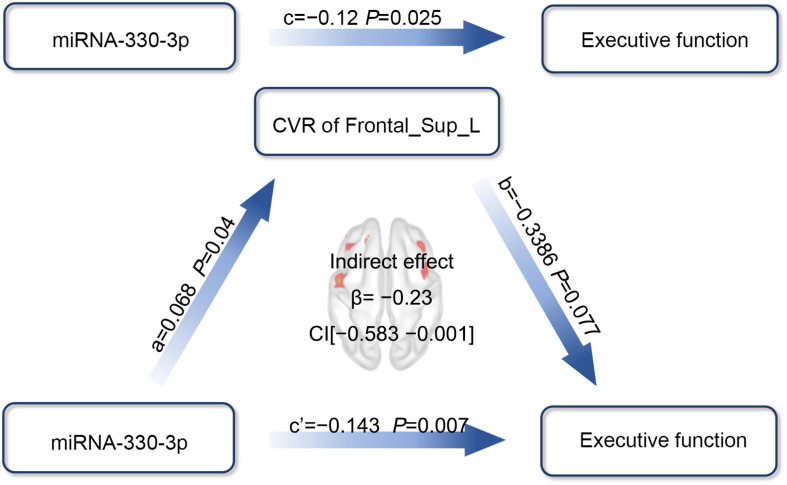
The mediation effect analysis of CVR in the left medial superior frontal lobe on the association between miRNA-330-3p and executive function in HT-CI patients. The standard coefficient and *P*-value were displayed on the pathway controlling for age, years of education, and history of lacunar stroke. The indirect mediation effect of *β* = –0.23 and a 95% CI as (–0.583, –0.001) was present in the center of the diagram. Red block indicated the significant CVR region. Frontal_Sup_Medial_L, left medial superior frontal gyrus.

When the effect of CVR on the association between the exosomal miRNAs and CI was investigated, we used the factors that showed significant correlation to build path models. We previously demonstrated that both miRNA-330-3p and CVR in the left medial superior frontal lobe were significantly correlated with executive function, and miRNA-330-3p was associated with CVR. Thus, we analyzed whether the CVR in the left medial superior frontal lobe mediated the association between miRNA-330-3p and executive function, controlling for age, years of education, and history of lacunar stroke. The association between miRNA-330-3p and executive function was mediated by CVR in the left medial superior frontal lobe (indirect effect: −0.023; 95% CI of bootstrap: −0.0583, −0.001; total effect: −0.1427, 95% CI of bootstrap: −0.2450, −0.0404) (Figure 7).

## Discussion

The major findings of the present study were as follows. HT-CI subjects displayed several decreased miRNAs (miRNA-330-3p, miRNA-432-5p, and miRNA-625-3p) and increased miRNA-6852-3p in serum exosomes, most of which were related to inflammation and proliferation pathways. HT-CI subjects also displayed significantly lower CVR in the left frontal regions than HT-NC subjects and healthy subjects. Correlation analysis showed that both exosomal miRNA-330-3p level and the CVR in the left medial frontal gyrus were negatively correlated with executive function in HT-CI subjects. Notably, the CVR in the left medial frontal gyrus mediated the association between miRNA-330-3p level and executive function. These findings furthered the understanding of mechanisms underlying the development of CI in hypertensive patients.

Previous studies have detected a variety of abnormal miRNA expression in hypertensive patients. Li et al. (2011) found that 18 miRNAs were decreased and nine miRNAs were increased in the plasma of hypertensive patients, and one of the miRNAs, human cytomegalovirus (HCMV)-encoded miRNA, hcmv-miR-UL112, was independently associated with the risk of hypertension. Hijmans et al. found that circulating expression of miRNA-34a was increased and the expression of miRNA-21, miRNA-126, and miRNA-146a were decreased in hypertensive patients. Furthermore, the levels of miRNA-34a, miRNA-21, miRNA-126, and miRNA-146a were associated with blood pressure (Hijmans et al., 2018). The heterogeneity of abnormal miRNAs has been shown by previous studies. These abnormal miRNAs are mainly involved in inflammation, angiogenesis, renin-angiotensin-aldosterone system, nitric oxide release, and ROS production, and most of the pathways are related to endothelial function (Nemecz et al., 2016). Thus, these abnormal miRNAs may eventually lead to endothelial dysfunction in hypertensive patients. The present study explored exosomal miRNA levels in hypertensive patients and found abnormal exosomal miRNAs related to hypertension and CI. These abnormal miRNAs, including decreased miRNA-330-3p, miRNA-432-5p, and miRNA-625-3p and increased miRNA-6852-3p, in HT-CI subjects, were mainly related to inflammation and proliferation pathways. Notably, the results were displayed by hypertensive patients with MCI, suggesting that these abnormal miRNAs played a major role in the onset of both hypertension and hypertension-related CI. The findings also suggested that although miRNAs contributed to hypertension through many pathways, the inflammation and proliferation pathways may be more related to hypertension-related CI.

The CVR pattern in hypertensive patients has been investigated by studies using transcranial Doppler or fMRI technique. Traditionally, transcranial Doppler is useful for measuring global CVR. Decreased global CVR was shown in both children and adolescents with hypertension and was associated with increased diastolic blood pressure (Wong et al., 2011). Elderly people with hypertension also displayed decreased global CVR, which was associated with lower executive function scores (Hajjar et al., 2014). These findings suggested that global CVR reflected the diastolic blood pressure and might predict the onset of CI in hypertensive patients. On the other hand, fMRI technique has been used to detect the regional CVR. Decreased CVR has been shown across different cortical regions, including the frontal cortex, parietal cortex, and cingulum cortex, in hypertensive patients (Savoia et al., 2011; Haight et al., 2015). In the present study, CVR differences were detected not only between hypertensive patients and healthy subjects but also between hypertensive patients with MCI and those with normal cognition. Both HT-NC subjects and HT-CI subjects displayed lower CVR in the left precentral gyrus than healthy subjects. Compared with HT-NC subjects, HT-CI subjects also displayed lower CVR in the frontal regions, including the left prefrontal white matter, the left inferior frontal gyrus, and the left medial frontal gyrus. The results suggested that the impaired cerebral vasodilatory activity of hypertensive patients was prominently shown in the frontal lobe, and the impaired vasodilatory activity played an important role in the development of CI related to hypertension. Furthermore, compared with healthy subjects, HT-NC subjects displayed increased CVR in the frontal regions, suggesting that compensatory vasodilatory activity in the frontal lobe might happen before the onset of CI in hypertensive patients.

Generally, vascular CI is characterized by impaired executive function, information processing, attention, and visuospatial function (O’Brien et al., 2003). Deficits in these domains are related to vascular lesions within the frontal lobe and basal ganglia regions, reflecting impairments of the frontal–subcortical loop (Román et al., 2002; Hsu et al., 2016). A great deal of evidence supports the hypothesis that the frontal cortex coordinates the transfer of various perceptual information from contextual cues and executes corresponding behavioral responses to achieve specific goals, which is known as the perception–action cycle (Fuster, 2001; Miller and Cohen, 2001). While many studies found that fMRI activity in the frontal cortex was associated with multiple cognitive performances (Wierenga et al., 2008; Obler et al., 2010; Gu et al., 2019; Liu R. et al., 2019), the present study extended these associations to regional vasodilatory activity and cognitive performance, i.e., the CVR in the frontal regions was significantly associated with executive function, visuospatial function, and language. Thus, not only the brain activity but also the vasodilatory activity in the frontal regions could reflect the development of CI in hypertensive patients. Notably, both exosomal miRNA-330-3p level and the CVR in the left medial frontal gyrus were negatively correlated with executive function in HT-CI subjects, and the CVR mediated the association between miRNA-330-3p level and executive function. To the best of our knowledge, the present study was the first to show the mediation effect of CVR on the association between miRNA and cognition in hypertensive patients. The mediation of CVR may be explained with the pathology of vascular CI.

Previous studies indicated that the expression of miRNA-330 was significantly decreased in human prostate cancer, melanoma skin cancer, and colorectal cancer, which inversely correlated with its direct target specificity protein 1 (Sp1), E2F1 transcription factors, and thymidylate synthase (TYMS) expression. After overexpression of miRNA-330 in these cancer cells, cell growth, migration, and invasion capability were suppressed (Lee et al., 2009; Mao et al., 2013; Xu et al., 2017; Sehati et al., 2020). However, miRNA-330 overexpression promoted cellular proliferation of glioblastoma through directly targeting the 3′UTR of SH3GL2 gene (Qu et al., 2012). A recent study reported that miRNA-330 overexpression in Alzheimer’s disease contributed to the reduction of amyloid β, oxidative stress, and mitochondrial dysfunction by targeting VAV1 through the MAPK pathway (Zhou et al., 2018). In addition, downregulated miRNA-330 could reduce myocardial infarction size through suppressing left ventricular remodeling (Liu Z. Y. et al., 2019). The discrepancy in miRNA-330 biological effects may result from the different diseases, tissues, and experimental conditions used. In the current study, we found that the HT-CI group had lower miRNA-330-3p than the control group and HT-NC group. Besides, the results of KEGG pathway analysis showed that many inflammation and proliferation-related pathways were significantly enriched in HT-CI subjects. The abnormal inflammation and proliferation could be accompanied by massive release of prostanoids and vascular endothelial growth factor, which accelerated vascular leakage, protein extravasation, and cytokine production. These processes disrupted endothelial function and reduced cerebral blood flow through various cerebral arteries, reflected by the decreased CVR in hypertensive patients. Specifically, the deep penetrating arteries supplying cortical projection fibers, association fibers, and subcortical nuclei were more susceptible to be damaged than other arteries. Then, the brain activity and connectivity were disrupted and the CI happened in hypertensive patients. The potential mechanism of miRNA-330 in the pathogenesis of hypertension-related CI needs further investigation.

Several limitations of the present study should be addressed. Firstly, the present study was a single-center, retrospective cohort study design. In consideration of the heterogeneity of the miRNA and the CVR in hypertensive patients, our findings should be validated with a larger sample from multiple centers. Secondly, the approach of resting-state BOLD applied in measuring cerebrovascular disease based on the finding that global BOLD signal within the frequency of 0.02–0.04 Hz has a significant correlation with end-tidal (Et) CO_2_ (Liu et al., 2017). However, it is possible the signal contains interference from some non-CO_2_ material. Finally, as the miRNA levels varied in different stages of hypertension, further experiments were needed to detect the expression levels of miRNAs at different time points.

## Conclusion

In conclusion, hypertensive patients with MCI displayed abnormal exosomal miRNA expression and decreased frontal CVR, both of which were related to the CI in these patients. The mediation of the decreased CVR on the association between exosomal miRNA and CI revealed the link between exosomal miRNA, cerebral vasodilatory activity, and CI in hypertensive patients. The decreased frontal CVR and the decreased exosomal miRNA-330-3p might become early biomarkers for the CI related to hypertension.

## Data Availability Statement

The datasets presented in this study can be found in online repositories. The names of the repository/repositories and accession number(s) can be found below: https://www.ncbi.nlm.nih.gov/sra/PRJNA685950.

## Ethics Statement

The studies involving human participants were reviewed and approved by the Drum Tower Hospital Research Ethics Committee. The patients/participants provided their written informed consent to participate in this study.

## Author Contributions

JM, XC, and QY contributed to conduct and analysis of the study, and writing of the manuscript. FC, RQ, and YC contributed to conduct the study. XZ contributed to concept and design of the study and conduct the study. YX contributed to concept and design of the study, and review and editing of the manuscript. All authors contributed to the article and approved the submitted version.

## Conflict of Interest

The authors declare that the research was conducted in the absence of any commercial or financial relationships that could be construed as a potential conflict of interest.

## Footnotes

1 http://rfmri.org/DPABI
