# Supplementary figures and images for: Exosomal MicroRNAs Contribute to Cognitive Impairment in Hypertensive Patients by Decreasing Frontal Cerebrovascular Reactivity
Source: Front Neurosci. 2021 Mar 1;15:614220. doi: 10.3389/fnins.2021.614220 (PMC7957933; doi:10.3389/fnins.2021.614220)

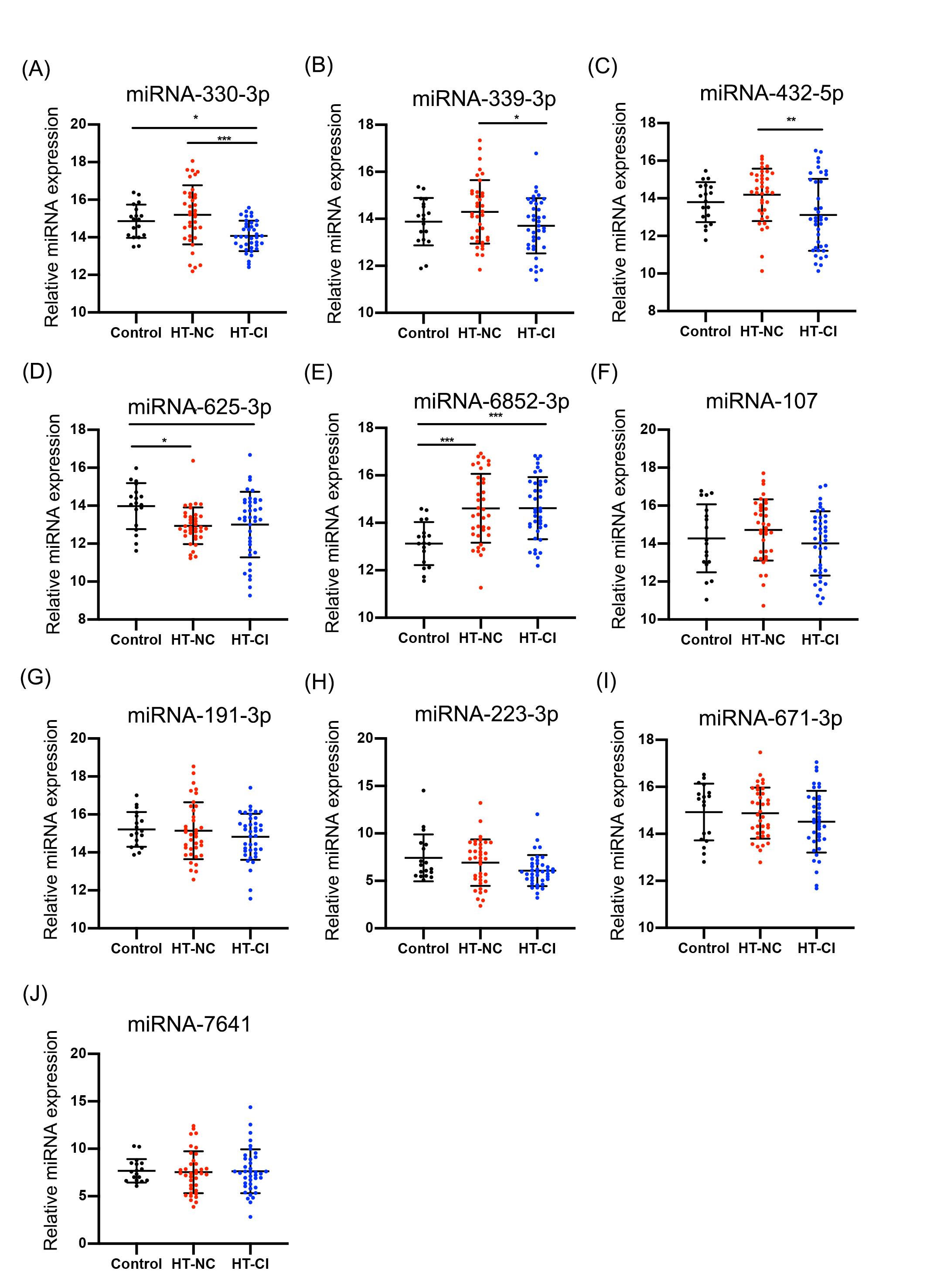

Supplement: Supplementary file 1 [file Image_1.TIF]
