# Supplementary material for: Exosomal MicroRNAs Contribute to Cognitive Impairment in Hypertensive Patients by Decreasing Frontal Cerebrovascular Reactivity
Source: Front Neurosci. 2021 Mar 1;15:614220. doi: 10.3389/fnins.2021.614220 (PMC7957933; doi:10.3389/fnins.2021.614220)
Supplement: Supplementary file 2 [file Table_1.DOCX]

Supplementary Material

## Supplementary Figures

**Supplemental Figure 1. Relative expression level of 10 exosomal miRNAs in patients and healthy controls.** **(A)-(E)** The relative expression level of miRNA-330-3p, miRNA-339-3p, miRNA-432-5p, miRNA-625-3p and miRNA-6852-3p. Values are the mean ± SD. **(F)-(J)** There is no significant difference of miRNA-107, miRNA-191-3p, miRNA-223-3p, miRNA-671-3p, miRNA-7641 between HT-NC, HT-MCI and NC groups. Error bar indicated standard error. ^**^*P*<0.01, ^***^*P*<0.001.
